# The (Real) Neurogenic/Gliogenic Potential of the Postnatal and Adult Brain Parenchyma

**DOI:** 10.1155/2013/354136

**Published:** 2013-02-06

**Authors:** Luca Bonfanti

**Affiliations:** Neuroscience Institute Cavalieri Ottolenghi (NICO), University of Turin, Regione Gonzole 10, 10043 Turin, Italy

## Abstract

During the last two decades basic research in neuroscience has remarkably expanded due to the discovery of neural stem cells (NSCs) and adult neurogenesis in the mammalian central nervous system (CNS). The existence of such unexpected plasticity triggered hopes for alternative approaches to brain repair, yet deeper investigation showed that constitutive mammalian neurogenesis is restricted to two small “neurogenic sites” hosting NSCs as remnants of embryonic germinal layers and subserving homeostatic roles in specific neural systems. The fact that in other classes of vertebrates adult neurogenesis is widespread in the CNS and useful for brain repair sometimes creates misunderstandings about the real reparative potential in mammals. Nevertheless, in the mammalian CNS parenchyma, which is commonly considered as “nonneurogenic,” some processes of gliogenesis and, to a lesser extent, neurogenesis also occur. This “parenchymal” cell genesis is highly heterogeneous as to the position, identity, and fate of the progenitors. In addition, even the regional outcomes are different. In this paper the heterogeneity of mammalian parenchymal neurogliogenesis will be addressed, also discussing the most common pitfalls and misunderstandings of this growing and promising research field.

## 1. Introduction

The discovery of neural stem cells (NSCs) at the beginning of the nineties led many people to consider definitively broken the dogma of the central nervous system (CNS) as made up of nonrenewable elements [1–3]. This finding, along with the characterization of adult neurogenesis in the olfactory bulb and hippocampus [3–5] triggered new hopes for brain repair. Yet, twenty years after, we realize that the dream of regenerative medicine applied to brain/spinal cord injuries and neurodegenerative diseases is still very far [6, 7]. As a matter of fact, adult neurogenesis in mammals occurs mainly within two restricted areas known as “neurogenic sites” [3, 8]: the forebrain subventricular zone (SVZ), reviewed in [9]; and the hippocampal dentate gyrus (subgranular zone, SGZ), reviewed in [10]. As a direct consequence of such topographical localization, most of the CNS parenchyma out of the two “classic” neurogenic sites remains substantially a nonrenewable tissue. An indirect proof of this statement resides in the fact that most of the traumatic/vascular injuries and neurodegenerative diseases, which actually occur in “nonneurogenic” regions, have still not found efficacious therapies capable of restoring CNS structure and functions through cell replacement. Thus, two decades after the discovery of NSCs and the reaching of a satisfactory characterization of adult neurogenic sites, a gap remains between the occurrence of stem/progenitor cells in the CNS of adult mammals and their effective capability to serve in brain repair. Several aspects do converge in explaining this gap [11] and, partly, in accounting for the heterogeneity of CNS structural plasticity in mammals (summarized in Table 1). During the last decade, new heterogeneity has been revealed by studies showing a substantial and widespread gliogenic [12–16], and to a lesser extent, neurogenic potential [17–19] within the CNS parenchyma, namely, in those areas previously considered as nonneurogenic. This new field of investigation revealed many unexpected potentialities for *de novo* cell genesis in the CNS, although most aspects of parenchymal neurogliogenesis remain quite obscure and ill-defined. In particular, several unresolved aspects make parenchymal neurogenesis a difficult territory to be explored: (i) the contrast between a wide range of potentialities displayed by parenchymal progenitors isolated *in vitro* and far more restricted potentialities which can be observed *in vivo* [20, 21], (ii) the existence of studies reporting neurogenesis in parenchymal regions which have been denied or not confirmed by other researchers [22–24], and (iii) the real origin of progenitors which are induced to proliferate/migrate in different lesion models (either mobilized from neurogenic sites or activated locally within the parenchyma; see Boxes 1 and 2) [25–28].

**Figure 1:**
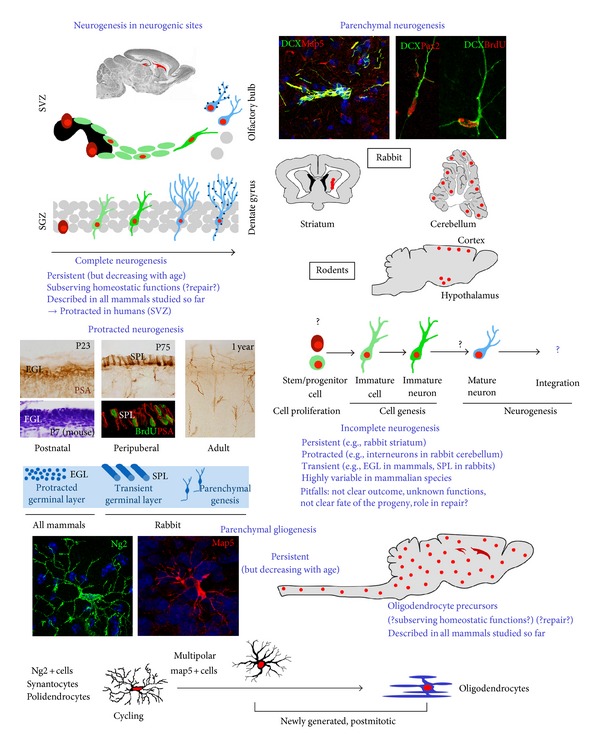
Heterogeneity of cell genesis (neurogliogenesis) in the mammalian CNS. Schematic summary of the features and location of different neurogenic and gliogenic processes occurring spontaneously in the postnatal and adult CNS. Red dots indicate newlyborn cells. SVZ: subventricular zone; SGZ: subgranular zone; EGL: external germinal layer; SPL: subpial layer (rabbit); PSA: PSA-NCAM; P23: postnatal day 23. Question marks indicate lack of knowledge about the origin, late differentiative steps, and final integration of newly generated parenchymal neurons. Photographs: top left, cluster, and chain of neuroblasts in the adult rabbit striatum (courtesy of Paola Crociara); top right, newly generated neurons in the adult rabbit cerebellar cortex (modified from Ponti et al., [19]); middle, modified from Ponti et al., [35]; bottom, Ng2+ polydendrocyte and multipolar Map5+ cell in the adult mouse and rabbit, respectively, (courtesy of Paola Crociara).

In this paper the *in vivo* neurogenic/gliogenic potential of the mammalian brain parenchyma will be analyzed with particular reference to variables involved in its heterogeneity (see Figure 1 and Table 1). In order to avoid one of the most common misunderstandings, namely, the confusion between occurrence of *de novo* cell proliferation in the CNS tissue and existence of true gliogenic/neurogenic processes (see Box 1), the attention should be focused on the outcome(s) of the newly generated progeny [32].

Before addressing in detail the heterogeneity of mammalian CNS structural plasticity and cell genesis, a brief summary of comparative adult neurogenesis and progenitor cell developmental origin will be given. Indeed, evolutionary explanations can provide an understanding of the logic followed (or not) by neurogenic processes through phylogeny, also accounting for the failure in CNS repair/regeneration and scarce usefulness of adult neurogenesis as a possible solution for brain repair in mammals [42]. In parallel, since developmental changes account for loss of CNS reparative/regenerative capacities and neuro-glio-genic potential, it is also important to know the real origin of different types of progenitor cells.

## 2. A Comparative View of Adult Neurogenesis and Brain Repair

As a matter of fact, failure in mammalian brain repair after traumatic, vascular, and neurodegenerative injuries is due to (i) a substantial lack of CNS reparative/regenerative capacity, (ii) a strong reduction in the extension of neurogenic regions within the whole CNS, (iii) the fact that adult neurogenic sites serve specific physiological functions rather than brain repair; for review, see [11, 40, 43]. It is important to note that if the occurrence of good neurogenic potentials would generally favor brain repair (at least by making available stem/progenitor cells) there is not a direct, linear relationship between occurrence of stem/progenitor cells and repair/regeneration, the latter processes strongly depending on the tissue environment and/or tissue reactions (for selected examples of neurogenesis and regeneration see [41]). 

Comparative studies on adult neurogenesis in the animal world show that neurogenic processes are detectable in wide regions of the CNS in invertebrates and nonmammalian vertebrates [29, 44, 45]. By contrast, in mammals neurogenic processes are restricted to two privileged areas (neurogenic sites) and the remaining CNS is largely made up of nonrenewable tissue [32, 46, 47]. The state of substantial “general plasticity” and cell renewal existing in the oldest living metazoans, so that all cell types, including neurons, are balanced in their production and loss [48, 49], is progressively reduced in vertebrates, although fish and amphibians still maintain remarkable regenerative capacities [50, 51]. Then, in birds and mammals a transition between regeneration permissive and nonpermissive stages occurs soon after birth, and highly-restricted spots of adult neurogenesis serve homeostatic functions in specific neural circuits [52, 53]. The decrease in neurogenic abilities occurs in parallel with topographical/numerical restriction of germinal layer-derived stem cell niches, whereas the decrease in regenerative abilities occur in parallel with other aspects: the impossibility to re-access to embryonic developmental programs during adulthood [54], the lack of differentiated cells capable of dedifferentiation [55], the development of a strong immune surveillance [56] and the consequent tissue reactions which are detrimental (reviewed in [11, 41]). In some cases, the stem cells found in the CNS of nonmammalian vertebrates are deployed for postnatal development of parts of the brain until the final structure is reached. In other cases, postnatal neurogenesis continues into adulthood leading to a net increase of the number of neurons with age (reviewed in [45]). Finally, in other cases, stem cells fuel neuronal turnover. An example is the cerebellar granular layer, which actually functions as a protracted development in postnatal mammals, whereas it becomes a persistent neurogenesis in adult teleosts, by continuously growing so that no definite adult cerebellar size is reached [45].

In addition, when considering mammals, the failure in CNS repair is a result of evolutionary constraints in which the injured tissue would not favor a strategy of regeneration but rather one of minimizing further damage (e.g., gliotic reaction [57]). In conclusion, as a consequence of multiple converging aspects, CNS regenerative capacity in mammals could have reached a point of nonreturn, in parallel with the persistence of some neurogenic processes which remain mainly focused on physiological functions (e.g., cell renewal/addition in selective neural circuits linked to learning/memory tasks [52, 53]).

An increased consciousness of the fact that scarce CNS reparative capacity in mammals depends on multiple aspects should indicate that it is very unlikely that the finding of a single molecular factor or pharmacological treatment capable of eliciting repair/regeneration. Comparative results from vertebrate species of different classes have demonstrated that adult neurogenesis is widespread among vertebrates but is employed by different species in different functional contexts [53, 58, 59]. In addition, a growing number of reports show remarkable heterogeneity even among mammals [17–19]. This variability concerns both neurogenic sites and parenchymal neurogenesis (reviewed in [32]; see below). This fact, along with a still incomplete knowledge of adult neurogenesis in humans (especially within the parenchyma), partially hampers the reaching of well-established “common rules” which might be used in the translation of experimental preclinical data to human medicine. Hence, dealing with mammalian CNS structural plasticity, at least two levels of heterogeneity should be taken into account: that involving different “types” of neurogenic processes (addressed in the next paragraph) and that of interspecies differences (mainly developed in the paragraph on parenchymal neurogenesis). 

## 3. Different Types of Cell Genesis in the Mammalian CNS

Detailed investigations carried out on the cellular, molecular, and functional outcomes of “classic” neurogenic sites revealed they are consistently present in all mammals studied, although with some differences [32]. Particularly when the outcome(s) of the neurogenic process are involved, the differences could be remarkable. The occurrence of a rostral migratory stream which is active throughout life in rodents but temporally restricted to the postnatal period in humans [60] is a prototypical example of variability among mammals. Indeed, in humans this neurogenic process seems to fall in the category of delayed developmental processes rather than adult neurogenesis (see below). 

In addition to differences in neurogenic sites, studies carried out during the last two decades revealed the presence of local, parenchymal progenitors which retain some proliferative capacity in most of the mature mammalian CNS [12, 14, 15, 17–19, 61]. This fact suggests that structural plasticity involving *de novo* cell genesis in the CNS could be more widespread than previously thought, but also different when occurring in neurogenic sites or in the parenchyma (Table 2). As a consequence of the increasing number of reports investigating adult neurogenesis in mammals, our perception of this biological process has gained new perspectives and nuances (for deeper analysis see [29, 32–34]). What was previously thought as “the genesis of new neurons in restricted brain areas endowed with NSCs,” can now be intended as a highly heterogeneous phenomenon (summarized in Figure 1), whose heterogeneity depends on several variables (see Table 1). The main elements of heterogeneity can be summarized as follows: (i) the location of progenitors: gathered within restricted neurogenic sites or widely spread out in the parenchyma; (ii) the nature of the progenitors: *bona fide* NSCs versus different types of progenitors; (iii) the genetic and molecular features of the progenitors: cell lineage (neuronal-like versus glial-like); identification of differentiative stages (dependent on the available markers); (iv) the existence or not of well-characterized neurogenic niches: absence of niches or occurrence of atypical/non-identified niches in the parenchyma; (v) the extension in time after birth: protracted, transient, persistent neurogenesis; (vi) the ultimate fate of the progeny in terms of cell lineage: neuronal versus glial, astrocytic versus oligodendrocytic; (vii) the ultimate fate of the progeny in terms of cell integration into circuits: complete versus incomplete neurogenesis; (viii) the spontaneous occurrence of the process versus its injury-induced appearance (briefly addressed in paragraphs 5 and 6). This latter point could be considered a further step beyond the so-called “constitutive” neurogenesis, namely, the spontaneous, continuous genesis of new neurons as part of a physiologic, homeostatic process [62].

Due to multifaceted aspects of the above mentioned processes, some problems of terminology can also be raised (summarized in Box 1). A common misunderstanding consists of a different use of the word “neurogenesis”, which can be intended either as “genesis of neurons” or as “genesis of neural cells”, that is, neurons and glia. Embryonic neurogenesis, namely, the process of building up the whole CNS, involves both neuro- and gliogenesis, occurring in largely overlapping and strictly intermingled phases, whereas neurogenesis and gliogenesis can occur separately in the adult. The landscape is even more complex, since research on adult neurogenesis brought developmental neuroscience within the mature brain, and the intermix of structurally plastic changes involving cell genesis/differentiation with the fully assembled adult tissue is accompanied by a previously unexpected intermix of cell lineages (e.g., newly formed neuroblasts arising from astrocytic-like stem cells *in vivo*). For this reason, in this paper, when not speaking of well-characterized cell lineages, the notion of “cell genesis” instead of “neurogenesis” will be used, since in most “neurogenic” processes different cell types can be considered among the progenitors, and different progenies can be generated. Hence, apart from detailed knowledge gathered around the activity of SVZ and SGZ neurogenic sites, many aspects of parenchymal cell genesis remain obscure and/or unexplored, as a consequence of the heterogeneity depicted above. In the last few years, parenchymal neurogliogenesis was among the most studied—yet less known—issues, due to the widespread location of the progenitor cells and to the substantial lack of markers which specifically identify their real origin as well as the stage-specific steps of their differentiation. As a consequence, the presence/absence of neurogenic processes within different CNS parenchymal regions in different mammalian species is still quite controversial and debatable. In most cases, parenchymal cell genesis occurs at low levels, at the limit of technical detection. Furthermore, in some cases it is very difficult to show its final outcome(s), most of the parenchymal neurogenesis appearing “incomplete” as to the final differentiation/integration of the progeny [32] (Figure 1). Finally, to correctly classify both germinal layer-derived and parenchymal neurogenesis some other aspects should be taken into account, such as the temporal extension of “protracted”/“transient” developmental neurogenic processes with respect to a “constitutive”/“persistent” neurogenesis [32]. A further aspect is that of potential/lesion-induced neurogliogenesis, namely, the genesis of new cells as a consequence of different types of CNS injury [25, 26, 28, 63] or altered homeostasis [64]. The latter issue will not be addressed in detail in this paper mainly focused on spontaneous/physiological states.

## 4. Origin of Adult Neurogenic/Gliogenic Processes

One of the features making possible the remarkable neurogenesis occurring in the neurogenic sites is their direct origin from embryonic, germinal layers which retain stem/progenitor cells along with the “niche” environment allowing their activity [10, 65]. The SVZ and SGZ actually are remnants of their embryonic counterpart, from which they maintain several cellular and molecular aspects [9] in parallel with an adaptation to the changing anatomy of the postnatal and adult brain [66, 67].

During development, the CNS originates from the neuroepithelium, pseudostratified epithelial cells that maintain contact with both the ventricular and pial surfaces. As brain thickness increases, neuroepithelial cells transform into radial glia [65, 68]. Beside their classic role as scaffolding for migrating neurons during embryogenesis and their subsequent transformation into parenchymal astrocytes of the mature CNS [69, 70], more recent studies have shown that radial glia cells behave as stem cells, leading to the genesis of astrocytes, neurons [71, 72], and, to a lesser extent, oligodendrocytes [73]. Thus radial glia cells not only serve as progenitors for many neurons and glial cells soon after birth, but also give rise to adult SVZ stem cells that continue to produce neurons throughout life [73]. The origin of astrocytes that function as neural progenitors in the adult hippocampus has not been determined experimentally. A connection to radial glia cells has been suggested even in the hippocampal SGZ [74, 75]. The relationship of adult NSCs to their developmental precursors offers clues to the unique characteristics that distinguish these germinal astrocytes from other astroglial cells in the brain parenchyma [65]. Indeed, parenchymal astrocytes lose very early their stem cell potential (around postnatal day 10 in mice [76]), although they can still proliferate in the severe gliosis induced after lesion [77], and resume multipotentiality *in vitro* [37]. 

On the other hand, gliogenesis persists throughout the CNS in the form of parenchymal cell genesis capable of creating new oligodendrocytes and, to a lesser extent, astrocytes, throughout life [12, 15]. Most of this gliogenic activity is attributed to synantocytes/polydendrocytes (Ng2+ cells; see paragraph 6) which are widespread in the CNS and whose origin is still partially obscure. Oligodendrocytes originate from migratory and mitotic embryonic precursors which progressively mature into postmitotic myelin-producing cells. The sequential expression of developmental markers defines distinct phenotypic stages in the oligodendrocyte lineage, characterized by proliferative capacities, migratory abilities, and changes in morphology. Most knowledge on this issue comes from studies on the rodent embryonic spinal cord. The first oligodendrocyte-committed cell appears at embryonic day 12 (E12) in two columns in the ventral ventricular zone of the motor neuron progenitor domain [78], which is defined by the expression of Olig2 [79]. The embryonic oligodendrocyte precursors are identified by their expression of platelet-derived growth factor alpha receptor (PDGF*α*R) [80]. The appearance of the oligodendrocyte lineage-associated markers Olig2 (essential for oligodendrocyte specification and differentiation) and PDGF*α*R (which permits the expansion of the original precursor population) is dependent on the concentrations of Sonic hedgehog (Shh) [81, 82]. One or two days after their appearance, PDGF*α*R+ cells exit the ventricular zone and expand by local proliferation and migration first in the ventral spinal cord region and then dorsally [83]. Finally, they occupy the entire parenchyma by the time of birth [80]. A dorsal source of oligodendrocyte precursors was also shown to contribute to oligodendrogenesis in the spinal cord and hindbrain [84, 85]. Fate mapping experiments revealed a double source of oligodendrocyte precursors in the forebrain: cells expressing oligodendrocyte lineage markers, such as Olig1, Olig2, Sox10, and PDGF*α*R, first appear ventrally, in the neuroepithelium of the medial ganglionic eminence, and then migrate laterally and dorsally into all parts of the developing forebrain by E16 to birth [86]. However, several studies have provided evidence for a dorsal source of oligodendrocyte precursors in the lateral and/or caudal ganglionic eminence(s), which constitute a second wave of cells invading the cortex only by E18 [85, 87]. Nevertheless, adult oligodendrocytes derive only by dorsal precursors, since medial ganglionic eminence-derived precursors were demonstrated to completely disappear after birth [87]. On the whole, it is thought that a unique oligodendrocyte population can derive from progenitor domains defined by different signaling molecules, in contrast to what has been established for neuronal specification during embryonic development, where different parts of the ventricular zone generate distinct types of neurons. In the rodent CNS, once PDGF*α*R+ cells have left the ventricular zone, they start to be termed “oligodendrocyte progenitor cells” (OPCs) and acquire their most typical marker: an integral membrane chondroitin sulphate proteoglycan named Ng2 (nerve/glial antigen 2). Ng2 expression becomes detectable only at E14 [88], thus, from E17 to adulthood all PDGF*α*R+ cells are Ng2+, and, conversely, all the parenchymal (nonvascular) Ng2+ cells are PDGF*α*R+ [88, 89]. Early embryonic Ng2+/PDGF*α*R+ OPCs are small, undifferentiated, proliferative and motile cells [90]. During embryogenesis, their morphology changes rapidly from a simple oval or polygonal cell body with few unbranched processes to a more differentiated and branched shape with a smaller cell soma [88, 91].

Coming back to comparative adult neurogenesis, nonmammalian vertebrates including fish, amphibians, and reptiles harbor a more widespread genesis of neurons in the parenchyma. Such processes, due to their location, are apparently independent from the primitive germinal layers. Nevertheless, recent studies which analysed in more detail the origin of adult neurogenesis in fish show that all neurogenic processes likely originate from remnants of the germinal layers (reviewed in [45]). Teleost proliferation zones reflect a general proliferation pattern along the ventricular walls of the brain, distinctly localized in all its subdivisions along the rostrocaudal axis. Between 12 and 16 distinct proliferation zones have been recognized in different teleost species [45]. Hence, across different animal classes, most stem cell populations retain contact to the ventricular system, and they appear as neuroepithelial cells, radial glia, or astroglial cell types. The different shapes of these progenitors have been suggested to be a secondary consequence of the architecture of the developing parenchyma overlying the ventricular stem cell zone of the embryo [9]. This common pattern across animal species, along with data reported above on the origin of cycling glial progenitors in mammals, indirectly suggests that adult parenchymal neurogliogenesis ultimately derives from embryonic germinal layers, yet being able to persist independently in some cases. 

## 5. Parenchymal Neurogenesis

Spontaneous (constitutive) parenchymal neurogenesis can be considered as a very rare phenomenon in mammals, and its regional location has been shown to be dependent on the animal species, age, and physiological/pathological states [32]. Different examples of neurogenesis occurring outside the two neurogenic sites have been described in rodents [19, 61], rabbits [17, 18], and monkeys [92, 93]. Remarkable differences can be observed between closely related orders (e.g., rodents and lagomorphs [17, 18]), between species (e.g., rat and mouse [19, 23, 93, 94]), and even different strains [95, 96]. 

Most parenchymal neurogenesis described in adult rodents seems to occur spontaneously at very low levels, rather being elicited/enhanced after specific physiological or pathological conditions [19, 61, 63, 64] (see below). Dayer and colleagues [17] showed the occurrence of new neurons in the deep layers of the rat cerebral cortex. By labelling newlyborn cells with multiple intraperitoneal injections of 5-bromo-2′-deoxyuridine (BrdU) and using markers of both immature and mature neurons to characterize the new cells through a detailed confocal analysis at different survival times, they demonstrated genesis of new GABAergic interneurons in both neocortex and striatum. At 4-5 weeks survival time, the 0.4 +/− 0.13% of the BrdU+ cells were mature NeuN+ neurons in the neocortex. Morphologic and phenotypic analyses assert these cells belong to different categories of cortical interneurons. Interestingly, although several BrdU+/DCX+/Tuc4+ neuroblasts were identified close to the SVZ periventricular region, the great majority of cortical BrdU+ cells were positive for Ng2. From these data the authors suggested that adult cortical newborn interneurons might originate from *in situ* progenitors. Other examples of spontaneous parenchymal neurogenesis have been described in lagomorphs. In rabbits, newly generated neurons are spontaneously produced in other regions of the adult brain starting from local, parenchymal progenitors. In the caudate nucleus, newly formed neuroblasts form longitudinally arranged doublecortin (DCX) and PSA-NCAM immunoreactive striatal chains similar to the SVZ chains [17]. These neuroblasts are generated from clusters of proliferating cells which express the astroglial marker brain lipid binding protein (BLBP), and about 1/6 of surviving cells differentiate into calretinin striatal interneurons. Always in rabbits, in sharp contrast with our common knowledge concerning the CNS of other mammals studied so far, a remarkable genesis of cells is detectable in the peripuberal, and to a lesser extent, adult cerebellar cortex [18]. Systemically administered BrdU detected at different postinjection survival times (up to two months) reveals newly generated PSA-NCAM+/DCX+/Pax2+ interneurons of neuroepithelial origin homogeneously distributed in the cerebellar cortex. Thus, in the striatal and cerebellar parenchyma of lagomorphs new neurons are generated independently from the remnants of germinal layers, yet their final outcome and their role in the adult neural circuits remains obscure; reviewed in [32]. 

The heterogeneity in parenchymal neurogenesis adds to that described for neurogenic processes occurring in adult neurogenic sites, which have been related to adaptation to ecological pressures [59]. At present, this is one of the most satisfactory functional explanations for adult neurogenesis in the entire phylogenetic tree, along with multiple, genetically determined variables spanning from the brain anatomy/developmental history to the animal lifespan [97]. This range of possibilities can also be increased by nongenetic variables, such as experience-dependent cues [58, 59].

Among the unsolved issues of parenchymal neurogenesis are the numerous reports which have not been confirmed by further studies or by other laboratories [22, 23, 26, 98–100], along with a series of data which have been denied in studies trying to reproduce the same results [24, 36, 101, 102]. Without entering in the scientific and technical discussion about these controversies, it is evident that we still did not grasp the real limits of parenchymal neurogenesis and that further studies are required before finally accepting, or denying the existence of some neurogenic processes. On the other hand, what appears clear is that some stem/progenitor cells in the parenchyma are able to give rise to new neurons in experimental and/or pathological conditions [28, 63, 64]. Various examples of “reactive” neurogenesis are known to occur after different types of CNS injury. Beside neurogenesis induced from adjacent neurogenic sites [25, 27], some neurogenic/gliogenic processes are also thought to start from local, parenchymal progenitors [28, 63, 103, 104]. For instance, local progenitors in layer I of the rat cerebral cortex, which in normal conditions seem to be rather quiescent, are activated after ischemia giving rise to new cortical interneurons [63]. Also in a slow and progressive model of striatal neuronal degeneration [105], besides activation of SVZ progenitors, genesis of neuroblasts has been found to occur also from local progenitors in mice [28]. This suggests that certain pathological states can stimulate either migration of progenitors from the adult SVZ or activation of local neuronal progenitors. Yet, one of the issues which remain poorly investigated is whether the adult brain parenchyma belonging to spontaneously nonneurogenic areas could be endowed with quiescent progenitor cells which can be stimulated to awake under specific environmental conditions, independently from the contribution of germinal layers. In other words, what remains irresolute is whether spontaneous and lesion-induced neurogenesis follow the same pathways and/or involve the same progenitors. Then, another intriguing possibility to be explored is that lesion-induced neuroblasts occurring in multiple forms of brain injury are committed to transient neuronal types [93], which contribute to restorative rather than replacement mechanisms [43].

A case placed in between the spontaneous and experimentally-induced neurogenesis is that of the hypothalamus. Several publications based on experiments carried out on rodents have been reporting data on this brain region as a new site for adult constitutive neurogenesis in mammals (for review see [106]). Under physiological conditions, both in rats [107] and mice [108, 109], proliferative activity does occur in the ependymal layer of the third ventricle and within the surrounding parenchyma. In rats, Xu and collaborators [107] using electron microscopy and immunohistochemistry showed that tanycytes lining the third ventricle proliferate and express molecules usually found in glial, stem-like progenitor cells, such as BLBP and nestin. The presence of putative neural progenitors was further supported by the isolation of cells able to give rise to neurospheres from the hypothalamus. One month after BrdU injection, proliferating cells, some of which expressing Hu protein, were detected in the surrounding parenchyma. Similar results were obtained in mice [108], yet in both rodent species no clear evidence has supported constitutive and complete hypothalamic adult neurogenesis under physiological conditions. A significant increase in hypothalamic proliferating cells can be obtained by performing i.v. delivery of BrdU (350% more positive nuclei, in comparison to i.p. treated animals), nevertheless, in spite of such cell proliferation the level of neurogenesis in the intact hypothalamus seems to be arrested at a very premature stage. On the other hand, growth factor infusion [61, 107, 110] or certain experimental conditions/models, such as prolonged heat exposure [111] and the AgRP-Tfam mutant mice (with Agouti-related peptide neuronal degeneration) investigated by Pierce and Xu [64], seem to increase neurogenesis in the hypothalamus. Intracerebroventricular infusion of insulin growth factor I in rats [110] triggered an intense proliferation along the third periventricular area and in the parenchyma of the caudal hypothalamus. As concerns the genesis of new neurons, after i.v. treatment with bFGF in rats [107] and CNTF in mice [61], it was shown that proliferation induced by growth factors can be followed by genesis of newborn neurons. Detailed morphological and molecular analyses of the third periventricular region of these animals showed interesting architectural similarities with the SVZ neurogenic niche (e.g., proliferating astroglial cells contacting the ventricle by an apical process bearing a single cilium), with tanycytes as primary proliferating elements lining the third ventricle [110]. Yet, additional studies are necessary to clearly demonstrate/confirm whether hypothalamic newborn neurons generated after physiological/pathological stimulation actually become part of the preexisting circuits playing a role in energy-balance mechanisms. 

Taking into account the multifaceted aspects dealing with parenchymal neurogenesis, difficulties encountered in such type of research are not only technical. They are also linked to the occurrence of processes placed in the middle between two well-characterized extremes of structural plasticity, such as synaptic plasticity and “complete” adult neurogenesis. In a recent review article [32] five levels have been dissected in the neurogenic processes in order to critically evaluate/compare different parenchymal neurogenic events (a graphic representation of the five levels is given in Figure 1). The subsequent steps span from cell division to possible integration of specified/differentiated elements into the CNS tissue, and according to this view, only when any of the five steps are filled the neurogenic process should be classified as “complete”. As a result, all the parenchymal neurogenic processes described until now can actually be considered as incomplete. This could explain why many claims of neurogenic processes were subsequently refuted because not sustained by experimental evidence. The piriform cortex is one of those regions in which results reported by different researchers are quite controversial; see, for example, references [92, 112–114]. Since long time, this cortical region is known to harbor a population of neurons immunoreactive for PSA-NCAM and DCX [114–116], which are two markers highly expressed in newly generated neurons but also present in nonnewly generated cells [116]. Indeed, deeper investigations have shown that the piriform cortex contains a population of immature, nonnewly generated neurons which display very few (or no) synapses and are frequently sheathed by glial lamellae [114]. These cells, by remaining in an immature state for indeterminate time, can represent a “reservoir” of neurons that could possibly be recruited into the preexisting neural circuits although not generated *ex novo* [117]. 

In conclusion, alternative and multiple forms of plasticity involving neurons can overlap within the so-called nonneurogenic tissue, affecting preexisting cells/circuits and increasing the complexity of the whole picture of brain structural remodeling. 

## 6. Parenchymal Gliogenesis

In the past, neurogenesis and gliogenesis had always been kept separate, the latter being considered less important than the former. In recent years, adult gliogenesis has been reevaluated as many populations of progenitor cells with glial-like features and proliferative capacity have been shown to exist in the mature mammalian CNS [13, 15]. As a matter of fact, parenchymal cell genesis in the so-called nonneurogenic regions is mainly gliogenic. In most regions of the CNS, parenchymal progenitors assure a slow process of “constitutive” gliogenesis leading to renewal of oligodendrocytes and, to a lesser extent, astrocytes [12, 15, 118]. In rodents, the major population of cycling progenitors located outside the germinal niches are Ng2+ cells morphologically, antigenically, functionally distinct from mature astrocytes, oligodendrocytes, and microglia [12, 14, 15]. These cells are also called “polydendrocytes” to highlight their stellate morphology and lineal relationship to oligodendrocytes [15], “synantocytes” [14] for their contiguity to neurons, or “oligodendrocyte progenitor cells” (OPCs) because they were found able of generating myelinating oligodendrocytes [12, 119, 120]. Nevertheless, many polydendrocytes remain as a resident cell population of Ng2-expressing cells in the mature white and grey matter after oligodendrocytes are generated. Thus it is widely accepted they represent the fourth CNS major glial population [15], representing 2–9% of total cells [13]. In the last decade, Ng2+ cells have generated a lot of interest among neuroscientists, because they show a series of features quite unusual in OPCs. These include (i) an almost uniform distribution in both grey and white matter; (ii) a stellate morphology; (iii) an intimate association with neurons from which they receive synapses [13, 14]; (iv) proliferative capacity in the adult brain [13, 121, 122] (v) a potential for giving rise to astrocytes and neurons that may be recruited to areas of lesion in the context of brain injury or pathology [118]. At present, it is generally accepted that polydendrocytes are OPCs, even if the demonstration that polydendrocytes differentiate into mature myelinating oligodendrocytes *in vivo* is challenging, because Ng2 expression is lost before the terminal differentiation of these cells and the appearance of mature oligodendrocyte antigens. Some observations provide circumstantial evidences for the oligodendroglial fate of polydendrocytes *in vivo*. For instance, they coexpress the PDGFR*α*, and during the first postnatal week, in the corpus callosum and cortex, they start expressing the immature oligodendrocyte antigen O4 [123]. Polydendrocytes also express the basic helix-loop-helix (bHLH) transcription factors Olig1 and Olig2, which are required for oligodendrocyte specification and differentiation as well as Sox9 and Sox10 transcription factors [122, 124]. Moreover, pulse-chase labeling of proliferating cells using BrdU revealed that the number of BrdU+Ng2+ cells decreases while that of BrdU+ oligodendrocytes increases over time [12, 125]. Cell-grafting experiments have shown that polydendrocytes give rise to myelinating cells when they are transplanted into an environment free of endogenous myelinating cells [126]. Recently, more direct evidence for the oligodendroglial fate of polydendrocytes was obtained from cell fate-mapping experiments using transgenic mice that express Cre recombinase (Cre) in Ng2-expressing cells or that express inducible Cre (CreeR), under the regulation of the Cspg4, PDGFR*α*, or Olig2 genes, which enable determination of the fate of polydendrocytes at a given time during development [99, 127]. These studies showed that oligodendrocytes continue to be generated in the mature brain.

Early cell-culture studies showed that OPCs purified from rat optic nerves differentiate not only into oligodendrocytes but also into process-bearing “type-2 astrocytes” in the presence of serum factors, which led to the concept of bipotential oligodendrocyte type-2-astrocyte (O-2A) progenitor cells [38]. There are now controversial observations suggesting that bipotentiality of polydendrocytes might be real or an *in vitro* artifact [126, 128, 129], and most likely these cells are inherently capable of differentiating into astrocytes but are prevented from fulfilling their astroglial fate in the normal *in vivo* environment [118].

On the whole, while all of these studies consistently support the oligodendrocyte lineage of the Ng2+ cells, the genesis of astrocytes from Ng2+ cells is confirmed only during postnatal ages. All these different and sometimes controversial results may be explained by some methodological/technical differences, but may also reflect heterogeneity in progenitor cell populations/subpopulations (mostly not yet identified), which is far to be elucidated [36]. In this context, we have recently identified a population of multipolar glial cells immunoreactive for the microtubule associated protein 5 (Map5) [130], which share features but also differences with Ng2+ progenitor cells [18]. These multipolar, Map5+ cells are newly generated, parenchymal elements of the oligodendroglial lineage, which represent a stage-specific population of polydendrocytes (Crociara et al., in preparation; Figure 1). 

Another issue which remains unresolved not only for adult glial progenitors but also for parenchymal neurogenic progenitors, is their way of cell division. In other words, what it is still not clear is the real nature of adult CNS cycling cells in terms of stem or progenitor elements. Using lineage tracing by retroviral infection, BrdU labeling *in vivo,* and transgenic mice expressing tamoxifen-inducible Ng2creER and fluorescent Cre reporter alleles to study the fate of single Ng2+ cells has revealed that age and neuroanatomical location determine whether these cells can either self-renew, generate mature oligodendrocytes, or both [101, 131, 132]. Adult Ng2+ progenitors have a very long cell cycle and many of them can divide at least twice, only a limited proportion of the progeny differentiating into mature oligodendrocytes. After stab wound injury, many of these progenitors reenter the cell cycle very fast, whereas voluntary physical exercise shows the opposite effect with increased exit of the cell cycle followed by an enhanced and fast differentiation into mature oligodendrocytes [131]. Asymmetric division of Ng2+ cells has been recently shown to occur [133]. The Authors of this study observed that proteoglycan Ng2 segregates asymmetrically during mitosis to generate OPC cells of distinct fate, and a decrease in such asymmetry coincides with premalignant, abnormal self-renewal rather than differentiation. On the whole, the data available on proliferative dynamics of parenchymal progenitors still need further investigation but strongly suggest that adult CNS parenchymal cell populations are subject to profound modulation by environmental stimuli and can be involved in pathology. 

## 7. Concluding Remarks and Future Perspectives

A better knowledge of adult neurogenesis and gliogenesis and of its relative underlying mechanisms is considered fundamental in order to figure out new efficacious therapies for brain repair. Under pressure of this statement, studies on this topic have increased exponentially during the last two decades, sometimes leading to excessive emphasis about theoretical correlations between neuro-glio-genic processes and brain repair. Focusing on the “real” neurogenic/gliogenic potential of the mammalian CNS should avoid to turn an exciting biological discovery into a therapeutic illusion. The existence of NSCs opened the intriguing perspective of cell replacement-aimed therapeutic strategies for neurodegenerative diseases, yet, twenty years later, this approach is still hampered by overwhelming problems concerning the final integration of both transplanted and endogenously induced cells [6]. At the basis of this failure are evolutionary constraints [57] and the fact that cell renewal occurring in adult neurogenic sites is primarily involved in tissue homeostasis, being hardly useful in response to external injury and neurodegenerative brain damage affecting the parenchyma [11, 40]. In this context, the discovery of parenchymal cell genesis represents a new plastic potential to be explored within wide regions of the CNS, including those areas affected by different neurodegenerative diseases and traumatic injuries. Nevertheless, a vast number of reports currently published in the domain of parenchymal cell genesis (references in [32]), although accurate and carried out with multiple technical approaches, do suggest that in most cases newly formed elements barely survive and do not fully integrate. In addition, the extreme heterogeneity of parenchymal neurogliogenesis makes the mammalian CNS parenchyma a harsh territory for neuroscience studies and for brain repair, in which new unanswered questions are continuously opened (see Box 2).

## Figures and Tables

**Box 1:**
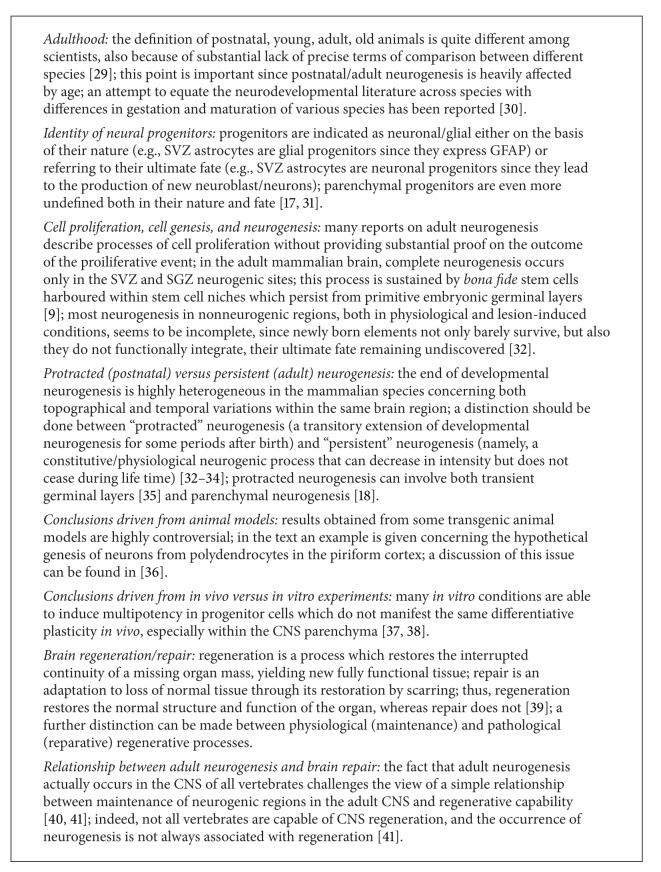
Major pitfalls and misunderstandings in adult neurogenesis concepts and terminology.

**Box 2:**
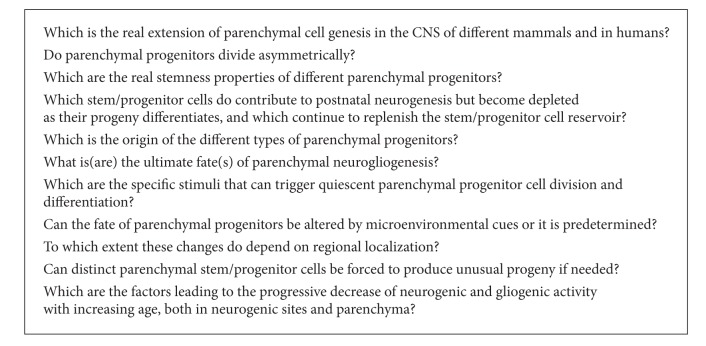
Some open questions.

**Table 1:** Variables affecting the nature and features of adult neurogenesis.

Animal species (animal world)	General plasticity and persistent neurogenesis are usually reduced across phylogeny; in parallel, the reparative/regenerative potential is also reduced
Animal species (mammals)	Unlike previous belief and current bias, remarkable differences in the location and extension of adult neurogenesis do exist among mammals
Age	Some neurogenic processes are extensions of delayed developmental programs (postnatal/protracted neurogenesis) whereas others persist throughout life (persistent neurogenesis). All neurogenic processes are progressively reduced with age
Microenvironment (niche)	A well-defined neural stem cell niche sustains neurogenesis in neurogenic sites (SVZ, SGZ), whereas a niche has not been characterized in parenchymal neurogenesis
Origin of stem/progenitor cells	Neurogenic sites (SVZ, SGZ) directly derive from persistence and modification of preexisting, embryonic germinal layers, whereas for parenchymal cell genesis such direct link is not clear
Location in the CNS	Location either within a germinal layer-derived niche or in the parenchyma redirects to the two previous points; in parenchymal neurogenesis many variations are linked to local cues of the different CNS regions involved
Function	In physiology: linked to the different ecological niches of the animals (present in all animals)
	In repair: linked to the species; in invertebrates and nonmammalian vertebrates the physiological function is associated with function in repair, whereas in birds and mammals it is only linked to physiology/homeostasis of specific systems

**Table 2:** Main differences between cell genesis in adult neurogenic sites and in the parenchyma.

	Neurogenic sites	Parenchyma
Location	Restricted	Widespread
Primary progenitor cells	Stem cells	Progenitors
Microenvironment	Stem cell niche	Mature parenchyma
Origin	Germinal layer derived	No direct link with germinal layers
Fate (progeny)	Mainly neurons (some astrocytes and oligodendrocytes)	Mainly glial cells (some neurons)
Fate (process)	Complete	Incomplete
